# Molecular Interactions between Pathogens and the Circadian Clock

**DOI:** 10.3390/ijms20235824

**Published:** 2019-11-20

**Authors:** Sailen Barik

**Affiliations:** 3780 Pelham Drive, Mobile, AL 36619, USA; barikfamily@gmail.com

**Keywords:** circadian clock, bacteria, virus, immunity, infection, host–pathogen, BMAL1, sleep cycle

## Abstract

The daily periodicity of the Earth’s rotation around the Sun, referred to as circadian (Latin “circa” = about, and “diem” = day), is also mirrored in the behavior and metabolism of living beings. The discovery that dedicated cellular genes control various aspects of this periodicity has led to studies of the molecular mechanism of the circadian response at the cellular level. It is now established that the circadian genes impact on a large network of hormonal, metabolic, and immunological pathways, affecting multiple aspects of biology. Recent studies have extended the role of the circadian system to the regulation of infection, host–pathogen interaction, and the resultant disease outcome. This critical review summarizes our current knowledge of circadian-pathogen interaction at both systemic and cellular levels, but with emphasis on the molecular aspects of the regulation. Wherever applicable, the potential of a direct interaction between circadian factors and pathogenic macromolecules is also explored. Finally, this review offers new directions and guidelines for future research in this area, which should facilitate progress.

## 1. Introduction

Sunrise and sunset bring us day and night in a 24-h cycle due to the rotation of the Earth around its own axis. In humans, visible light is detected by retinal photoreceptors, and the signal is transduced to the so-called “master clock” in the suprachiasmatic nucleus (SCN) in the hypothalamus [1]. In human society, a deranged circadian clock often manifests itself in a large range of sleep disorders [2]. However, the existence of an “endogenous” timekeeper that operates autonomously without any direct light signal was recognized early on and termed the “circadian clock”(CC). The CC acts as a biological oscillator or pacemaker with an approximately 24 h cycle, synchronized with solar day and night. While it was always obvious to the most casual observer that all animals follow the day and night cycle in their daily behavior—and the phototropism of plants was also extensively studied—the molecular underpinning of the CC began with the revelation that it is entrained in virtually every tissue of the body as well as in single cells. Thus, the CC is essentially cell-autonomous, functioning in a 24 h cycle, irrespective of light or darkness. The CC and its interaction with infectious agents constitute the main theme of this review.

Although this review is focused mainly on the central clock, it should be noted that electrical and endocrine signals from the central clock, which originate in the SCN, signal to and orchestrate another ‘clock’ set, known as the “peripheral clock” [3,4]. Present in the other regions of the brain and in the tissues of the body, the peripheral clocks are partly independent, as they are entrained in the individual tissues. Together the central and the peripheral clocks play an important role in the systemic physiology of the animal as well as in specific tissues [5], among which the skin and the lung are important for invasion by pathogens, as detailed later. The field of circadian biology has witnessed a flurry of studies from a large number of researchers, and several masterly reviews have been published in recent times; for the sake of brevity, I have cited several comprehensive reviews along with recent new discoveries, as and where appropriate. I apologize to the many scientists whose original early research papers were not directly referenced.

## 2. The Mammalian Circadian Clock

**Figure 1:**
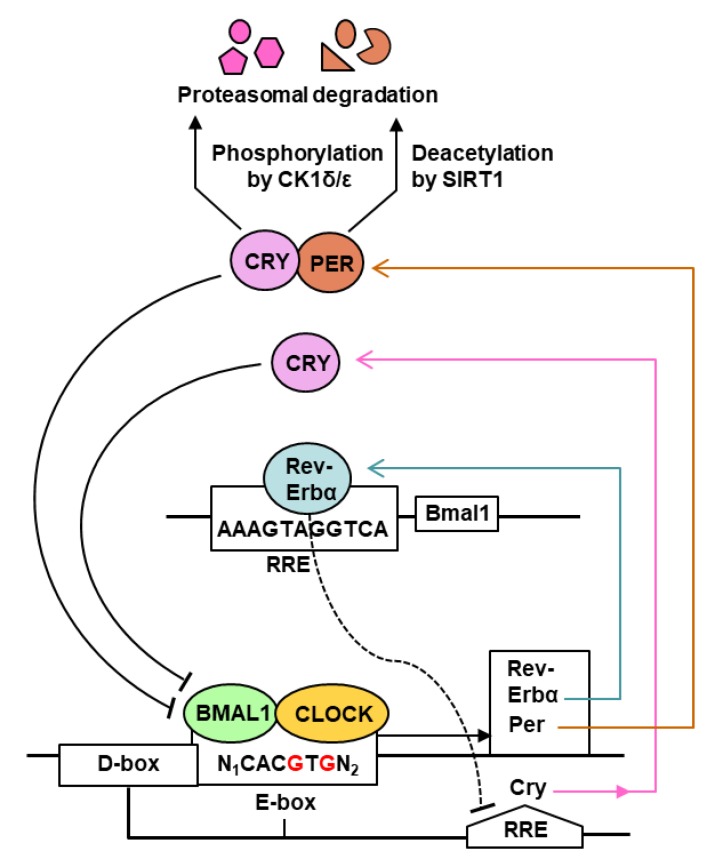
Feedback loops (TTFLs) of the mammalian circadian clock. This is a simplified snapshot of the highly complex circadian clock (CC) [2,3,4,5,6,7,8,9], depicting only the core pathways and the central players in transcriptional control. Most of the translational steps, including translation regulation by BMAL1 [10,11], are not shown for simplicity, but some are mentioned in the text of Section 2. As shown here and detailed in Section 2, the mammalian cell-autonomous 24-h CC is regulated by the core transcriptional activators (BMAL1, CLOCK) and two families of repressors (PER1, PER2 and CRY1, CRY2). BMAL1 and CLOCK function as a heterodimer [8] that binds to the E-box enhancers of the CC genes, such as *Rev-Erbα*, the *Per* and the *Cry* genes. Multiple other enhancers fine-tune several of these promoters, only a few of which are shown. The PERs and CRYs also heterodimerize and translocate to the nucleus (not shown), where they interact with and inhibit BMAL1-CLOCK. REV-ERBα binds RRE elements, inhibiting further transcription of *Bmal1* and *Cry* genes. Finally, PER and CRY proteins are degraded by the 26S proteasome, triggered by deacetylation and/or phosphorylation [12,13]. Broadly speaking, PER and CRY synthesis is activated by BMAL1-CLOCK as the day progresses, which then feedback-inhibits their own transcription. At night, PER-CRY is degraded; as a result, BMAL1-CLOCK activates a new round of PER-CRY transcription. However, none of the changes are instantaneous, as each biochemical output rises and falls in a gradual pace. For example, it takes several hours for a gene to produce optimal amounts of mRNA, and that mRNA population to be fully translated. Similarly, the PER and the CRY proteins have their own half-lives, and therefore, the population is degraded gradually, not instantly.

The core regulatory network of the mammalian circadian clock (CC) consists primarily of a transcription–translation feedback loop (TTFL) [6,7], in which transcriptional regulation plays a major part (Figure 1).

As shown (Figure 1), the CC network in mammals, including humans, is controlled by two activators, the basic helix–loop–helix transcription factors, CLOCK and BMAL1 (Brain and Muscle ARNT-Like 1, also known as ARNTL1, for Aryl hydrocarbon Receptor Nuclear Translocator-Like protein 1), and the repressors, the PERs (Periods 1 and 2) and the CRYs (Cryptochromes 1 and 2). CLOCK and BMAL1 form a heterodimer that binds E-box motifs in the promoter region of the CC target genes [8,9,10]. Interestingly, CLOCK possesses histone acetyltransferase activity that contributes to chromatin-remodeling events implicated in CC-regulation of gene expression [14]. The major gene products include negative regulators that repress CLOCK/BMAL1 activity, notably REV-ERBα (an orphan nuclear receptor, also known as NR1D), PERs, and CRYs. Other enhancers also contribute to achieve optimal transcription of each gene; the *Cry* gene, for example, is regulated not only by E-box (morning element), but also by D-box (day element) and RRE (evening element) [13]. As a rule, each enhancer recruits its specific transcription factor: the E-box recruits BMAL1-CLOCK, the D-box requires the D-box-binding protein (DBP), and the RRE binds REV-ERBα. In the negative part of the cycle, PERs and CRYs heterodimerize and translocate to the nucleus; at the same time, CLOCK acetylates BMAL1, which facilitates the interaction of the PER-CRY heterodimers with BMAL1-CLOCK, inhibiting further transcription. In addition, REV-ERBα negatively regulates several genes, notably those of *Bmal1* and *Cry*, by binding to their promoter RRE elements. In the post-translational segment of the CC, the PER and the CRY proteins are deacetylated by SIRT1 deacetylase and/or phosphorylated by casein kinase 1, marking them for degradation by the 26S proteasome [12,13]. Overall, the positive and negative feedback loops are orchestrated to repeat in a continuous 24 h rhythm, which forms the basis of the circadian periodicity [15,16,17]. In the translational segment of CC (not shown in Figure 1), the cytosolic BMAL1 protein stimulates translation upon phosphorylation by ribosomal S6 protein kinase, which increases its interaction with the translation machinery [10,11]. In addition, light-induced entrainment of the master CC in the SCN of the hypothalamus is promoted by phosphorylation of the translation initiation factor eIF4E, which specifically stimulates translation of *Per* mRNAs [18].

All evidence suggests that the classic CC network is the ‘tip of the iceberg’, and as such, new players and signaling branches are being routinely discovered. In an example of the intricacy, whereas PERs are phosphorylated by CK1 (Figure 1), this step is reversible because phospho-PER is dephosphorylated by PP1 [19], and PP1 itself can be regulated by association with one of many regulatory subunits [20]. Regulation of phosphorylation of CRY appears to be even more complex, as CK1 is autoinhibited by self-phosphorylation, which is relieved by the phosphatase PP5, while CRY noncompetitively inhibits PP5 [21,22]. A tetratricopeptide repeat (TPR) protein, PP5 is uniquely stimulated by the binding of polyunsaturated fatty acids to its TPR domain [23,24], which opens the possibility that CRY can be regulated by fatty acid metabolism. It has been reasoned that the hallmark complexity of the CC is necessary because a system composed of a small number of molecules would be intrinsically noisy, whereas one consisting of a larger number can be expected to be robust in the face of noise [25].

While much is now known about the CC of mammals, evidently due to the use of the laboratory mouse, how the CC of a host organism may interact with an infectious agent is a relatively recent query. Accumulating evidence has now clearly documented an interplay between the circadian clock of the host and the ability of a pathogen to infect as well as the severity of the disease state caused by the infection. Not unexpectedly, much of the interaction is also influenced by the immune status of the host, which in turn is often regulated by the CC, since the CC is present in immune cells as well.

In this review, the term “pathogen” is used to mean all kinds of infectious agents, namely, bacteria, parasites, fungi, or virus, regardless of whether or not they cause an overt disease. By the same token, all types of interaction have been included, regardless of whether they are mutualistic (i.e., both parties receive some benefit), commensal (one party benefits, the other is apparently unharmed), or parasitic (one party benefits, the other is harmed, often killed). This is based on the presumption that all interspecies interactions serve a purpose in the bigger context of nature, and therefore, have a place “under the Sun” and hence, in this review. As promised in the title, the main focus here is on the molecular mechanism wherever one has been deciphered. For introductory literature, the readers may consult earlier reviews that described the circadian phenomenology of such interactions from a classical biological or immunological perspective [26,27,28,29,30,31].

## 3. Clock-Controlled Genes (CCGs)

**Figure 2:**
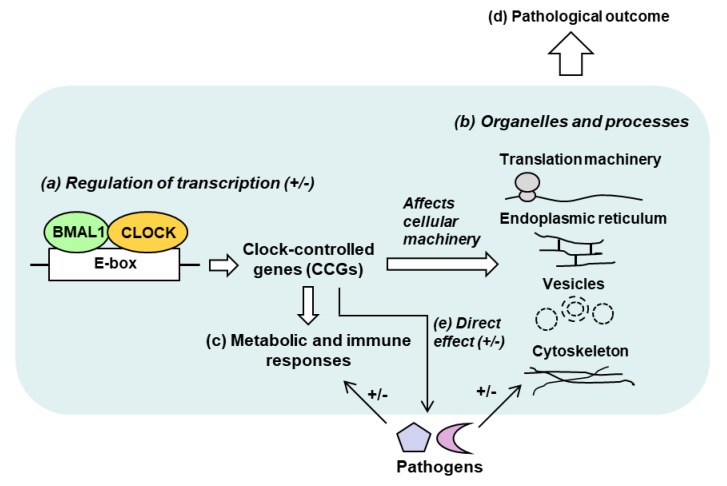
Schematic diagram of known and potential host-parasite interactions, relevant to the circadian clock. This is only a snapshot of the major systems in the proposed “Circadian–Metabolic–Microbial” Supersystem, described in greater detail in Section 7 and elsewhere. In its canonical role (**a**), the circadian BMAL1-CLOCK heterodimer transcriptionally activates a large number of clock-controlled genes (CCGs), although some other genes may be negatively regulated (denoted by +/−) [32,33,34,35,36,37,38,39,40]. The CCG products are structurally and/or functionally involved in modifying the cellular infrastructure and processes, such as specific organelles and ribosomes (**b**). They also affect metabolic pathways, aging and immunity (**c**). Together, these effects regulate the host’s response to infection positively or negatively (more or less susceptible to infection) [43]. The interaction can be reciprocal (not shown), i.e., the pathogen may also alter the clock, or suppress immunity [44]. The totality of these interactions affects the end result, which is the pathology and the disease, caused by the infection (**d**). Finally, circadian genes themselves (e.g., BMAL1, CLOCK and others) or the CCGs can more directly participate in pathogen replication (**e**). These have been elaborated in appropriate sections.

The advent of genome-wide expression analysis has begun to unravel the clock-controlled genes (CCGs) that are candidate effectors of the circadian cycle. Several studies in the past and in the recent times performed transcriptome analyses of diverse tissues, harvested at different times, and from various clock-knockout (KO) mutant strains of mice; some also conducted large-scale polysome profiling of the transcriptome to determine the translational aspect of the CCGs [32,33,34,35,36,37,38,39,40]. In genetic analyses, the effect of knockout or mutations of the major clock genes have been studied in live mice or in cultured cells, as detailed later. Collectively, the results have revealed the following:

(i) Large number of CCGs: In any given tissue, ~10–20% of genes can be CCGs, i.e., genes regulated by the CC negatively or positively [33,34,35], which in human corresponds to a few thousand genes. (ii) Multiple classes: The large number of CCGs are distributed over a plethora of protein families and functional classes. Predictably, they also belong to diverse pathways and intracellular locations, including but not limited to lipid and xenobiotics metabolism, extracellular matrix, chemokine signaling [41], endoplasmic reticulum, cytoskeleton, protein localization, and intracellular vesicles [42]. (iii) Tissue specificity: The identities of the CCGs significantly differ between tissues, as seen for liver [33], lung [40], and skeletal muscle [35], suggesting that even within the same animal, the CC has specifically evolved for the optimal metabolism of each tissue and organ, which should be relevant to the tissue-tropism of pathogens. A broad schematic of host–pathogen interactive network in the circadian context is presented in Figure 2.

While much progress has been made in our understanding of the clock components and the identity of the CCGs, it is also apparent that direct studies of pathogens in the circadian setting are still in their infancy. Phenotypic studies of clock mutations have traditionally focused on general biology and physiology, and found a plethora of abnormalities that appear to belong to three main categories: Metabolic syndrome [45], aging, and immune dysregulation (Table 2 in reference [46]). Although much of this knowledge was derived from studies of knockout mice [46], the signaling routes connecting the central circadian clock to the peripheral clock [3,4,5] remained largely uncharted. The metabolic syndrome, referred to above, can indeed result from desynchronization between the central and peripheral clocks, for example through altered timing of food intake and diet composition [47]. Thus, wherever applicable in this review, I will explore the premise that the metabolic disorders that result from circadian dysfunction, are also likely to facilitate infections that are commonly seen in the traditional “metabolic syndrome” states (such as in type II diabetes and obesity), and in old age and immune deficiency.

## 4. Interactions between the Circadian Clock and Microbial Infection

There are two fundamentally distinct aspects of infection by cellular microbes that have the potential to be regulated by the CC of the host and/or the microbe: successful infection, and the outcome of the infection. While the first is mainly a consequence of host-seeking by the vector, the second is regulated by innate immunity. There are examples of both aspects, some of which will be covered in this Section.

### 4.1. Circadian Clock and Infection by Bacteria

The monocellular (unicellular) eukaryotes and prokaryotes comprise of major disease agents to the metazoa, but their chronobiology has remained rudimentary, mainly due to our understanding that most bacteria are not photoreceptive (the notable exceptions being the cyanobacteria and the purple bacteria). Indirect evidence of CC in non-photosynthetic bacteria has been reported sporadically in several well-known species such as *Escherichia coli*, *Klebsiella* sp., *Pseudomonas putida*, and *Bacillus subtilis*, based on growth measurements, swarming response, and reporter gene expression in laboratory assays [48,49]. Genome survey of all prokaryotes found homologs of the cyanobacterial circadian gene KaiC in several other bacteria, including the nitrogen-fixing rhizobacteria and *P. putida* [48]. However, the periodicity of several of the observed rhythms were shorter than 24 h, and thus, could not be strictly designated as circadian. Thus, these bacteria appear to possess a non-circadian rhythm, regulated by novel proteins, which may nonetheless affect their interaction with animals with CC, in a phase-separated manner. Indeed, several members of these aforementioned genera are responsible for acute human infections, in which the CC can play a role. Bacteria such as *E. coli* and *Klebsiella* sp. are common gut inhabitants, and part of the natural mammalian microbiome [50]. However, toxic strains of *E. coil* cause severe diarrhea, urinary tract infections, and acute kidney failure in children [51,52]; similarly, *Klebsiella pneumonia* can get outside the intestine and cause pneumonia, bloodstream infections, wound sepsis, and meningitis [53]. *Pseudomonas* is ubiquitous in the environment, and is also a common inhabitant of human skin; however, it also causes infections during hospital stay [54]. These infections are more likely to occur, and with more severe outcomes, in individuals with lower immunity, such as the elderly, the children, and those receiving immunosuppressive treatment to prevent tissue rejection [55,56]. Although circadian studies of these infections are lacking, such studies are warranted because a relationship can be anticipated for a multitude of reasons. First, many of the affected sites implicated above are part of the CC network, with their own tissue-specific clock gene expression profile and peripheral clocks [3,4]. The ability of *E. coli* and *Klebsiella pneumonia* to escape the gut microbiome (see below) should be affected by alteration of innate immunity during the day and night cycle, and also by sleep disturbances that are common in the elderly, the sick, and the caregivers working in night shifts, who are also exposed to nosocomial infections. Second, many common medications, including cardiovascular drugs, prescribed in metabolic syndromes can perturb the sleep cycle [57,58], which may in turn lead to lower immunity and higher susceptibility to the otherwise friendly resident bacteria. Much of our knowledge of host-microbe interaction derives from studies of the natural microbiome at various locales of the body, such as the lung and the gastrointestinal tract (GI), two areas of fervent research [50,59,60].

The relationship between the healthy mammalian host and its GI flora is mutualistic, in which both parties are benefited. While the microbes use the gut as their habitat and feeding ground, the humans use many products of bacterial metabolism, such as short-chain fatty acids and vitamins, and receive protection from invading pathogens [61,62,63]. In fact, severe disturbance of the gut flora, resulting from oral antibiotic treatments and various diseases, is generally associated with nutritional deficiencies and inflammatory conditions [63,64]. Disruption of the sleep–wake cycle in jet-lag travels has been shown to transiently affect the gut microbiome [65,66,67,68,69,70,71,72,73,74,75,76,77,78]. The interaction between CC, immunity, and microbes are discussed in detail in Section 6.

In an interesting recent development, melatonin, a CC-related hormone that is produced by the pineal gland in the brain and regulates the sleep–wake cycle, has been shown to affect the magnitude of swarming of *Enterobacter aerogenes*, an enteric bacterium that contains a homolog of the human melatonin receptor [69]. The effect is specific, as *E. coli* and *Klebsiella pneumoniae* are not affected. This is an example where a CC-related hormone, secreted externally, modulates microbial behavior in its vicinity. Since motility is a major contributor to a bacterium’s ability to access and infect host tissues, such results have important clinical implications. Due to its involvement in synchronizing the CC, melatonin is sometimes used to treat short-term sleep problems, such as resulting from jet lag or nightly work shifts [70]. Its observed effect on the gut microbiome now calls for an interrogation of its possible effect on the GI defense system and a re-evaluation of its physiological benefit.

A relatively unexplored area in clock studies is the attainment of competence to take up exogenous DNA, which is important in the transfer of plasmids or chromosomal DNA fragments between two cells. Best studied in *Bacillus subtilis*, competence was shown to be controlled by a set of excitable positive and negative feedback loops of regulatory proteins that are expressed in rhythmic fashion [71]. Since competence has the potential to disseminate new traits, such as antibiotic resistance between bacteria and genetic exchange between pathogenic bacteria and hosts, its circadian and seasonal variation can add new dimensions in host–pathogen interaction and in broader biology and evolution.

### 4.2. Role of Circadian Rhythm in the Transmission of Parasites and Fungi

The vast majority of parasites are transmitted by vectors such as birds and arboreal insects, which have their own circadian rhythms, thus creating a tripartite circadian interaction: Vector, parasite, and host. The Chagas disease in South America is caused by the protozoan parasite, *Trypanosoma cruzi*, which is transmitted by insects of the *Triatominae* subfamily, such as *Triatoma infestans* and *Rhodnius prolixus*, nicknamed “kissing bugs” [72,73]. As the principal vectors of *T. cruzi*, the diurnal behavior of these insects has received extensive attention. In both species, the CC seems to control multiple aspects of their biology, such as foraging, host-finding, egg hatching, and reproduction. Both insects, *R. prolixus* in particular, thrive in foothills of different altitudes, at temperatures ranging from 16 °C to 28 °C. However, as the CC is temperature-compensated, it is reasonable to assume that the 24 h periodicity is maintained in all locations. Both *R. prolixus* and *T. infestans* encode clock proteins that are orthologs of the fruit fly (*Drosophila*) clock proteins, PER and TIM, and are expressed rhythmically [72]. Quantification of the *Per* transcript in the neurons of *T. infestans* showed that the levels peak at dusk, consistent with the nocturnal activity of the insect [72,73]. As the parasite is transmitted when the vector insect bites its victims at night, the insect CC directly participates in disease spread. Although CC has not been studied in *T. cruzi*, it has been characterized in *Trypanosoma brucei* [74], the other epidemiologically significant parasite of the *Trypanosoma* genus, which causes the African “sleeping sickness”, a neuropsychiatric syndrome. Like *T. cruzi*, *T. brucei* is also transmitted by an insect, namely the tsetse fly (tsetse or tzetze means ‘fly’ in a native African language). In vitro, ~10% of *T. brucei* genes, mostly those enriched in metabolic pathways, were found to be expressed in a circadian rhythm [74]. Interestingly, the sensitivity of the parasite population to suramin, a drug commonly used for first-line treatment of sleeping sickness, also varies in a circadian manner [74]. These results not only demonstrate the existence of an intrinsic circadian clock in *Trypanosoma* that is independent of the host, but also offer the prospect of using anti-parasitic drugs at optimal times. In the natural setting, the combination of the CC of the *Trypanosoma* and its vector insects likely offers a composite CC-based phenotype for optimal parasite transmission.

Besides the Trypanosomes, several other unicellular protozoan parasites are transmitted by vectors that are predominantly nocturnal. Notable ones with a circadian connection are *Plasmodium* sp., the malaria parasite, which is transmitted through the bite of Anopheles mosquito, and *Leishmania major*, the agent of Leishmaniasis, transmitted by female sandfly bites [75,76]. By virtue of the nocturnal nature of the vectors, transmission of these parasites from vector to human occurs mainly at night and is, therefore, subject to the dark cycle of the CC. The cyclical nature of *Plasmodia* infections in producing malaria is an integral multiple of ~24 h. Studies in mice, using the rodent malaria parasite, *P. chaubadi*, suggested that synchronization with host rhythm influences parasite growth in the animal as well as the transmission potential of the parasite, thus contributing significantly to the evolution of host-parasite interactions [77].

On a more direct level of interaction, the trypanosome presents a fascinating example of a parasite altering the CC of the host to a certain extent. Several studies have documented that severe trypanosomiasis leads to disruption of the sleep–wake cycle in humans [78], likely due to parasitic infection of the SCN, the master circadian pacemaker of the brain. In infections by virulent strains, such as *T. brucei gambiense*, a major human pathogen, the infected SCN has been shown to undergo significant neural damage [79]. The cellular and molecular details of the parasite’s effect on the SCN and how it affects the host CC should be a revealing area of study.

The recurring fungal and yeast infections in the human population are represented by aspergillosis [80], caused by *Aspergillus* sp., and candidiasis [81], caused mainly by *Candida albicans*. Circadian clock in fungi is well established, mainly from genetic and phenotypic studies in *Neurospora* sp., which identified several major circadian proteins, named ‘Frequency’ (FRQ) and White Collar-1 and -2. As with the mammalian CC, the *Neurospora* clock proteins, although they are divergent from their mammalian counterparts, also operates in a feedback loop (TTFL) that involves protein–protein interaction and protein phosphorylation [82,83]. Motif analysis revealed that orthologs of these proteins are conserved in other fungal species, albeit to different degrees [83,84]. It is fair to assume that as in bacteria, the circadian clock of the pathogenic fungi will also interact with the rhythm of the mammalian host in infection, transmission, and immune response. However, detailed studies in this area are lacking, perhaps due to the slow kinetics of fungal infection and measurable pathology, which takes much longer than the 24 h time frame of circadian periodicity.

In conclusion, cumulative literature offers evidence that host–pathogen interactions for all three cellular pathogens, viz., bacteria, fungi, and parasites, can be influenced by the CC through multiple mechanisms, including the influence of the CC on the transmitting vectors. Moreover, all can be also influenced by the interactive immunity of host. Unlike the bacteria, the fungi and the parasites, for being eukaryotic in nature, appears to have their own CC, which is still poorly characterized. Regardless, an informed knowledge of the CC-based regulation of all three pathogens may offer novel strategies and drug regimens for their control.

## 5. Circadian Clock and Infection by Viruses

Viruses are molecular complexes that are obligatory parasites, which must infect cells in order to multiply, and as a result, do not have a circadian rhythm of their own. However, viruses are likely the most abundant parasites, found in almost every ecosystem on Earth, and for nearly every species and cell type. In view of the dependence of nearly every step of the virus life cycle on the host machinery and the large-scale regulatory effect of the CC on cellular gene expression, as mentioned earlier (Section 2 and Section 3), it is anticipated that virus replication will be affected by the host cell CC, although this remains to investigated at the cellular level. At the organismic level, virus-induced pathology and mortality have been found to exhibit circadian variation. Several such interactions were reviewed recently [44], although their molecular mechanisms have remained mostly unclear. A few mechanistically diverse examples are presented below.

The relationship between the circadian clock of the liver and the hepatitis C virus (HCV), which has a single-stranded RNA genome, has been studied extensively, revealing multiple interactive mechanisms. A positive-sense single-stranded RNA virus of the *Flaviviridae* family, HCV is the eponymous agent of hepatitis C and some forms of liver cancer (hepatocellular carcinoma) and lymphomas in humans [85]. In one study, HCV replication and the clock genes appeared to be mutually inhibitory, as HCV infection decreased PER and CRY expression, and overexpression of PER decreased HCV replication [86]. A large class of small non-coding RNAs, known as microRNA (miRNA), generally inhibits translation of mRNAs containing miRNA-complementary sequence at the 3′-untranslated region. However, in an opposite functionality, miR-122, an abundant liver-specific miRNA, was shown to interact with the 5′-terminal sequence of HCV genome and actually required for optimal HCV multiplication [87]. It was, therefore, logical to ask if miR-122 levels change in a circadian manner which could offer a molecular mechanism for the circadian behavior of HCV. Indeed, the levels of miR-122 precursor transcripts was found to oscillate ~5-fold in a 24 h period and that the miR-122 gene promoter contains functional REV-ERBα-binding elements [85]. Reciprocally, the amplitude of the oscillation was blunted in Rev-Erbα KO (knockout) mice. While these results offered initial hope as a possible mechanism, the level of mature miR-122, unlike that of the precursor transcripts, was essentially constant at all times, due to the long half-life of miR-122, very likely exceeding 24 h [88].

An alternative mechanism was discovered in a very recent study using the HCV-permissive Huh7 cells of hepatocyte origin in cell culture [89]. In this study, BMAL1 and REV-ERBα were shown to affect HCV growth in opposite ways: BMAL1 acted positively, and REV-ERBα negatively. It was also revealed that two major components of the cell-surface receptor of HCV, namely CD81 tetraspanin and tight junction protein claudin-1 [90], showed circadian expression in synchronized Huh-7 cells (Figure 1e), with the peaks coinciding with that of BMAL1 and with HCV uptake. Thus, both receptor genes are in fact members of the CCG family, and are induced by the BMAL1-CLOCK heterodimer. This was confirmed by silencing BMAL1 expression with CRISPR, which significantly lowered the levels of both receptors and strongly reduced HCV entry. In contrast, activation of REV-ERBα by SR9009, a specific agonist, inhibited HCV through a different pathway. Whole genome microarray analyses resulted in the identification of several thousand genes that were up- and down-regulated by upon treatment of Huh7 cells with SR9009. Pathway analysis of the differentially expressed genes showed an enrichment of metabolic pathways involved in lipogenesis and cholesterol/bile acid metabolism. SR9009 treatment specifically reduced the promoter activity of the stearoyl-CoA-desaturase (SCD), and its mRNA and levels, also causing a significant reduction in unsaturated fatty acids levels. Independently, CRISPR-mediated knockdown of SCD lowered HCV growth. Lipid and cholesterol are known to be essential for the replication of flaviviruses [88]. Together these results suggested that the inhibitory effect of REV-ERBα on HCV is mediated through two pathways: inhibition of BMAL1-CLOCK, the positive regulator, thus lowering CD81 and claudin-1, and inhibition of lipogenesis. Importantly, essentially similar results were obtained with two other flaviviruses, namely dengue and Zika viruses [89,90,91]. Thus, circadian control of flaviruses may co-opt conserved mechanisms, involving similar receptors and lipid moieties.

Recent reports have also demonstrated direct roles of the host circadian components in the replication of several other pathogens [42,43,89,92,93,94]. Three such studies [42,43,92] independently found that *Bmal1* -/- (knockout) mice supported enhanced growth and pathogenesis of three paramyxoviruses (respiratory syncytial virus, RSV; parainfluenza virus, PIV; Sendai virus, SeV) [43,92], an orthomyxovirus (influenza A virus, IAV), and herpesviruses, indicating an antiviral function of BMAL1. Similar virus-induced of pathology was also seen when the wild type animals were subjected to simulated jet lag [92]. The increased susceptibility of the knockout was not a result of an aberrant fetal development due to embryonic loss of *Bmal1*, as inducible post-natal deletion of the Bmal1 gene also led to enhanced SeV replication, and as a result, developed more extensive asthma-like airway changes [92]. Finally, enhanced replication of the viruses could be recapitulated in fibroblasts, isolated from the *Bmal1* -/- animals, as well as in wild type fibroblasts in which Bmal1 expression was silenced by transient transfection of specific short interfering RNA (siRNA) [43]. Together, these results clearly demonstrate a role of BMAL1 in cellular innate immunity against multiple viruses. While herpesvirus is a large double-stranded DNA virus that uses host cellular DNA-dependent RNA polymerase for its gene expression, the other viruses contain RNA genomes and encode their own RNA-dependent RNA polymerase. However, the innate immune function of BMAL1 was still specific in nature [43], since vesicular stomatitis virus, another RNA virus, showed the opposite phenotype, i.e., replicated efficiently in the wild type fibroblasts, but failed to replicate in the *Bmal1* -/- fibroblasts. *Toxoplasma gondii*, a monocellular protozoan parasite, grew equally well in the wild type and the *Bmal1* -/- fibroblasts [43]. Thus, the antiviral effect of BMAL1 appears to be relatively broad-spectrum, yet virus-specific, likely regulating common host factors that are co-opted by multiple viruses. Clearly, BMAL1 (and possibly REV-ERBα) serves as either a proviral or an antiviral host factor for different viruses, but in either case, it will serve as a mechanism for circadian effect on the virus growth.

The CLOCK histone acetyltransferase was shown to be an essential component of the herpes viral transcriptional machinery throughout the replicative cycle of the virus [93]. In some cases, the virus may reactivate. Epstein-Barr virus (EBV), another member of the large *Herpesviridae* family, formally called gammaherpesvirus 4, is the causative agent of mononucleosis, or “mono” for short, also nicknamed the “kissing disease” because of the way it can spread from one person to another [95]. Like herpes simplex, it remains latent in the body for years and can be reactivated to lytic replication [95]. Induction of the viral *Bzlf1* gene is one of the first steps in the reactivation of Epstein-Barr virus (EBV) [95], and changes in histone acetylation at the *Bzlf1* gene promoter appears to be a key part of the reactivation mechanism [94]. It is tempting to speculate that CLOCK-catalyzed histone acetylation is also capable of reactivating the *Bzlf1* promoter. Since the circadian protein levels cycle in rhythmically (Figure 1), these studies lay the foundation of mechanisms by which a circadian protein can regulate virus growth at different times of day and night by directly associating with a viral protein.

Virus transmission through infected donor organs is an unfortunate outcome of modern medicine [96,97] but lacks a clear understanding or prevention regime. Perhaps the best-studied example is liver transplantation, which is the only viable option in end-stage liver cirrhosis, often a result of chronic infection by HCV. Ironically, reinfection of the allograft is nearly universal so that the HCV cirrhosis returns and the viremia rebounds and even exceeds the pre-transplant levels. Immunosuppressive drug treatment, needed to prevent graft rejection, is partly to blame. In a recent investigation, reinfection subjects transplanted in the morning were found to have a significantly higher frequency of HCV rebound, compared to those transplanted in the afternoon, suggesting a circadian effect that deserves further investigation.

Reciprocally, viruses can also regulate the CC of the host. In representative examples, circadian proteins and HCV exhibited mutual antagonism: While expression of HCV core protein (genotype 1b) led to downregulation of PER2 and CRY2, overexpression of PER2 lowered HCV replication [86,98]. More recently, IAV has been shown to remodel the pulmonary clock in a murine model of chronic obstructive pulmonary disease (COPD): while chronic exposure to cigarette smoke combined with IAV infection altered the timing of clock gene expression and increased lung inflammation and emphysema, the effects were markedly elevated in *Bmal1* KO [99]. How the HCV core protein and a yet-unknown IAV function modulate the clock genes would be important questions to answer.

## 6. Circadian Clock in Intracellular Pathogen Growth and Cellular Immunity

Once a parasite enters the host system, it encounters a variety of defense mechanisms of the host, which determines the outcome and the severity of the infection and the resulting disease, which in turn affects the release of the progeny parasites and their transmission to the next round of hosts. Continued successful propagation in the population ensures a critical mass of the parasite, essential for its survival. The CC may be intimately involved in the regulation of any step of this cycle [27,28,29,30].

Early studies revealed that several aspects of the immune system exhibit circadian rhythms, much of which was recently reviewed in detail [27,28]. This included specific immune cells, such as monocytes, neutrophils, and lymphocytes, as well as the immunoregulatory cytokines, TNF-α and IL-6. The circadian rhythm of LPS-induced IL-6 elaboration was lost in *Bmal1* KO mice [100,101]. Secretion of TNF-α and IL-6 from LPS-stimulated wild type macrophages in culture also depended on the time, and exhibited circadian oscillation, which showed that the myeloid cells are innately clocked for immune response at the cellular level [102]. Consistent with a cell-intrinsic CC, the levels of the REV-ERBα transcription factor (Figure 1) of the macrophages also exhibited strong oscillations in a circadian manner. Finally, the afore-mentioned circadian response to LPS was lost in macrophages isolated from Rev-Erbα-deficient mice [28]. Similar studies showed that the circadian clock of the pulmonary epithelial cells also modulate immune responses to LPS or *Streptococcus pneumoniae*, which included elaboration of TNF-α, IL-6, and multiple CXC chemokines [101]. The CXCL5, in particular, was responsible for attracting neutrophils to the affected site, promoting the inflammatory response. Mice exposed to *Salmonella typhimurium* were infected to higher levels and developed more severe inflammation during the early rest phase, (e.g., 10 AM), compared with other times (e.g., 10 PM); this differential effect largely disappeared in *Clock* -/- mice [103]. These results convincingly connect the cell-intrinsic immune response to key molecules of the circadian clock (Figure 1); nevertheless, the molecular links that function between the clock and the target cytokines are still missing. To the extent that circadian disorder can lead to metabolic syndromes such as obesity and diabetes, the related loss of innate immunity [46,59] and susceptibility to bacterial infections may play an important role in public health. For instance, diabetic foot infections, an outcome of diabetic immunopathy, are caused mainly by several types of bacteria, including *Staphylococcus aureus* and *Enterobacteriaceae* [104].

*Leishmania major* is a prime example of a monocellular parasite using the CC of the host immune system [105]. Once introduced into the bloodstream of the victim through sandfly bite, *L. major* first infects neutrophils and is then transmitted to macrophages. What internalized by the phagocytes, the parasite hijacks host immune responses to its own advantage and thus evades innate immunity. As described earlier, most sandfly blood meals occur at night, and this led to investigations whether the parasite also takes advantage of the innate circadian rhythm of the host’s immune system for optimal replication and transmission. Indeed, in both footpad and peritoneal routes, *L. major* infection showed a circadian pattern over the 24 h cycle, paralleled by time of day-dependent neutrophil and macrophage infiltration to the infection site and chemokine expression [105]. Moreover, a similar cycle was also found for parasite replication in cultured mouse macrophages ex vivo. The circadian relationship was confirmed by the loss of all rhythms in macrophage cells isolated from Bmal1-KO mice [105]. In another mouse study of infection by the helminth, *Trichuris muris* [106], animals infected in the morning (i.e., rest phase) were observed to have lower worm burdens due to higher worm expulsion efficiency, compared to those infected in the late evening (i.e., active phase); the difference was lost in the *Bmal1*-KO mice. Expulsion efficiency correlated with a strong Th2 bias, and this was also lost in the BMAL1-deficient dendritic cells [106]. Interestingly, worm expulsion appeared to be related to feeding-driven metabolic cues, since feeding during the day in naturally nocturnal-feeding mice disrupted parasitic expulsion. Overall, these results indicate that the cell-intrinsic CC in the immune cells [106] also constitutes a major arm of host defense against parasite infection and growth.

**Figure 3:**
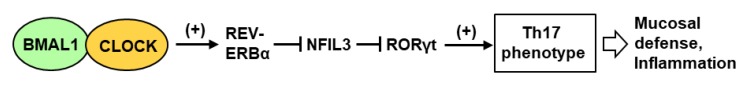
Cascade positive and negative transcriptional steps in clock-regulated IL-17-producing Th17 response [107]. The circadian clock BMAL1-CLOCK heterodimer transcriptionally activates the REV-ERBα gene (as in Figure 1). The REV-ERBα protein then represses the synthesis of the next transcription factor, NFIL3, which in turn represses the gene for RORγt, an orphan nuclear receptor, essential for Th17 cell specification.

Recent research has suggested a major contribution of the IL-17-producing T-helper (Th17) cells in the circadian immunity of the GI. In general, the Th17 cells are important in protecting mucosal surfaces from diverse pathogens, including bacteria and fungi, and their differentiation from CD4+ T cells was recently shown to be regulated by the CC [107]. In this regulatory circuitry (Figure 3), the lineage specification of Th17 cells is activated by the orphan nuclear receptor RORγt, the expression of which is negatively regulated by the transcription factor NFIL3 that represses the Rorγt promoter.

*Nfil3* gene transcription is repressed by REV-ERBα, the expression of which in turn is activated by BMAL1-CLOCK, a central circadian transcription factor (Figure 1). *Nfil3* gene expression, therefore, rises at night and drops during the day, whereas *Rorγt* gene expression follows the opposite pattern. The net result is that Th17 cell development is linked to the circadian clock network, and more specifically, Th17 cell differentiation occurs more readily from naïve T cells isolated during the day. This may also suggest that disruption of the light cycle, for example in jet-lag travels, would affect mucosal defense. In summary, although the natural microbiome does not cause an infection in a healthy skin or GI, the changing Th17 levels may alter its makeup, thus making the mucosa more or less resistant to pathogens at different times of day and night. The knowledge may facilitate better management of a person’s microbiome and timely avoidance of prospective foodborne and airborne pathogens.

In contrast to the nonspecific innate immunity, the vaccine-based acquired immunity is highly specific, and has been responsible for the containment and eradication of some of the most debilitating pathogens in human history, such as diphtheria, influenza, polio, pox, measles (rubella), and mumps, to name a few. Although there are preliminary clinical reports of better efficacy of vaccines when administered in the morning [108,109,110], they have not been followed through. If a circadian contribution to antigen response can be fully established, both its clinical benefit and mechanism would be motivating areas of evaluation and study.

## 7. Discussion and Conclusions

It should be clear from this short review that initial strides have been made in host–pathogen interactions in the context of the circadian clock, but much of the mechanism at the molecular level remains to be deciphered. Collective evidence suggests that at the organismic (whole animal) level, systemic physiological players, such as humoral and neural factors, control myriad aspects of metabolism and behavior, all of which can be affected by the CC. In turn, they can all regulate the host’s interaction with specific pathogens as well as immune response to the pathogens. The highly complex nature of circadian network and phenotypes often makes it difficult to ascertain whether a biochemical reaction is directly relevant to circadian rhythm or only distantly related to it, perhaps resulting from secondary effects such as immune or humoral responses that can be regulated by non-circadian factors. Nonetheless, as mentioned in various sections, direct effects have been forthcoming from recent studies using cell cultures, transcriptional reporter constructs, and biochemical assays To further facilitate an integrated view of this large multisystem network, the name “Circadian–Metabolic–Microbial” Supersystem was suggested earlier (Figure 2), in which the component systems can influence one another in any combination.

Much of the complexity of this supersystem arises not only from the many circadian regulators, but also from the vast network of the CCGs [34,35,36]. One of the first steps in understanding the network is to ask: Why so many CCGs? This question, posed either for the host alone or for host–pathogen interactions, has no clear answer. BMAL1, for example, is classically recognized as a transcription factor with a basic helix–loop–helix (bHLH) and two PAS (Per-Arnt-Sim) domains. The E-box enhancer, to which BMAL1 binds, has the palindromic consensus sequence, CACGTG; however, variants of this sequence and neighboring cis-acting sequences fine-tune BMAL1-binding [36,111]. Like most enhancer-dependent transcription factor, BMAL1 is, therefore, likely to regulate the transcription of multiple target genes in the mammalian genome (Figure 1 and Figure 3). In fact, in genome-wide profiling of BMAL1 was estimated to target >150 sites in the human genome, including all of the clock genes and many others [36]. Based on the common denominator of metabolic syndromes in circadian mutants [45,46], it is reasonable to assume that deficiency of the other clock functions, such as CLOCK, the PERs and the CRYs will also cause similar, if not identical, altered predisposition to pathogens. All of them may regulate a large set of common as well as specific CCGs, thus affecting multiple non-CC pathways in various combinations. The direct role of clock proteins in viral replication, free of the involvement of metabolic or immune pathways, is certainly an exciting area to microbiologists, but has gained momentum only recently, as illustrated by a few studies laid out earlier [42,43,89,92,93,94]. Evidently, a broader study of the role of clock genes in other viruses will unravel additional mechanisms.

In a largely untested scenario, the double-stranded DNA viruses in the Baltimore Group I (such as the herpes, papilloma, polyoma, pox, and adenoviruses, to name a few clinically important ones) may contain functional E-box elements [112], as these viruses share the nuclear DNA-dependent RNA polymerase II of the host. If so, these promoters, like those of the CCGs, may be activated directly by BMAL1-CLOCK, which is surely worth exploring. The same may be tested for retroviral DNA genomes that can remain latent, but requires transcription by host RNA Pol II for reactivation [113,114]. If so, these promoters, like those of the CCGs, may be activated directly by BMAL1-CLOCK, which is surely worth exploring.

A direct effect of circadian cycle on the viral promoter can be ascertained by real-time monitoring of expression of a reporter gene driven by the viral promoter. In a leading study [115], the immediate-early gene (*IE-1*) promoter of the human cytomegalovirus (CMV) was tested for response to light. CMV is a common virus in the population, and IE-1 expression is essential for its reactivation from latency. To test whether IE-1 can be under circadian control, bioluminescence was imaged in individual SCN cells from transgenic mice containing the *IE-1* enhancer/promoter upstream of firefly luciferase. A small percentage of the cells indeed displayed circadian transgene expression. As the authors concluded [115], single-cell bioluminescence imaging can be used to reveal that the circadian pacemaker can regulate exogenous viral genes and play a role in viral diseases.

Beyond transcription, the next level of complexity of the supersystem arises from translational and post-translational controls, which has been only briefly touched upon in this review. There is no doubt that future research will lead to the discovery of new layers of control. For example, as described earlier (Section 5), a role of REV-ERBα in reducing unsaturated fatty acid levels has been discovered very recently during studies of flavivirus growth [89], but we also know that CRY can be regulated by fatty acids via the protein phosphatase, PP5 (Section 2). The fatty acids, therefore, connect REV-ERBα and CRY in a novel away that has not been explored yet.

## 8. Suggested Guidelines for Molecular Circadian Studies

Lastly, I would outline a set of guidelines that can be taken into account in delineating the mechanism of a circadian event and its relationship with a pathogen. For the sake of simplicity, I have used a signaling phosphoprotein as a candidate player, but the criteria would apply for any other molecule, such as mRNA, transcription factor, protein kinase, or cytokine.

1. Multiple time points: The candidate molecular change, such as phosphorylation level, should be followed not just day and night, or in two points of ‘Zeitgeber’ (a German term for any environmental cue that entrains the biological rhythm to the Earth’s circadian cycle; often used in clock studies on laboratory animals in artificial light-and-dark cycles), but at multiple time points over at least 24 h, preferably longer [116]. For example, a candidate phosphorylation event should be analyzed by quantifying the phosphoprotein every 6 h, if not more frequently. This will reveal a rhythmic pattern, even if it is phase-separated from the oscillations of the pathogen or its vector.

2. Quantification: The amplitude of the cycle (often described as “fold-change”) should be determined by quantitative assays, such as densitometry, qPCR, and enzyme assays. Variation of amplitude from one peak to another may indicate new regulators appearing at various times, generating multiple temporal switches and consecutive phases. Such results have been demonstrated to underlie the robustness of redox oscillation [117].

3. Network assignment: In an exemplary time-series study, mRNA was purified at 2 h intervals over 48 h, which allowed a comprehensive modeling of multiple, co-existing transcription–translation loops, closed by delayed activation or repression [7]. In such a detailed analysis, the phosphoprotein of our example would be assigned to a specific loop, further validating its mechanistic association with a CC gene product. Proper assignment and pathway analysis are also important in genome-wide studies of biological rhythm [118].

4. Relevant cell types: As a rule, the choice of the laboratory animal is important for translation into human phenotypes. Mouse is a nocturnal animal, unlike most humans, and also have differences in immunology and pathology [119]. Evolution-guided functional analyses have recently revealed that the innate immunity-related *Ifit1* gene of mouse, which was long presumed to be equivalent to the human *Ifit1*, is actually not an ortholog [120]. In studying CC in cultured cells, one should ideally use the relevant cell type for tissue-trophic pathogens, such as gum cells for periodontal pathogens, lung cells for respiratory viruses, because the CC components may have tissue-specific rhythms [46]. It is now established that many cell lines that were traditionally considered epithelial, were in fact HeLa cells, which are fibroblasts [121], possible requiring re-interpretation of some findings.

5. Multiple animals and clones: Similar to the recommendation of using multiple animals in animal studies of CC, cell culture studies of a pathway should employ rhythm analysis in multiple clones. A recent study of 20 clock genes in a panel of 25 clones showed that variation in gene expression levels of at least five clock genes may underlie period-heterogeneity [122]. The variation was not stochastic, but rather heritable. Thus, the phosphoprotein cycle of each clone may best match the circadian rhythm of the same clone, but not another’s.

6. Multiple isolates of the pathogen: As with cells, multiple strains/varieties of the pathogen should be tested. The inherent cycle of the candidate phosphoprotein of the host may be shifted to a different cycle upon infection, and this may also vary between strains.

7. Dedicated algorithms: Recently developed algorithms, dedicated to the analysis of CC-regulated molecular network [123], can be employed to further validate the candidate and its relationship with other members of the network, whose functions may be known.

Reduction of the complexity to the cellular level has led to remarkable progress in our understanding of the molecular mechanism of cell-intrinsic CC. However, the real-life benefit of the studies must await an understanding of the total physiological response of the living animal, starting from the suprachiasmatic nuclei of the brain, which will require multidisciplinary and collaborative efforts.

## Abbreviations

BMAL1/ARNTL1	Aryl hydrocarbon Receptor Nuclear Translocator-Like protein 1
CC	Circadian clock
CCG	Clock-controlled gene
CRY	Cryptochrome
HCV	Hepatitis C virus
IL	Interleukin
KO	Knockout
LPS	Lipopolysaccharide
miR	MicroRNA
mRNA	Messenger RNA
PER	Period
PIV	Parainfluenzavirus
PP	Protein phosphatase
qPCR	Quantitative polymerase chain reaction
RSV	Respiratory syncytial virus
SCN	Suprachiasmatic nucleus
SeV	Sendai virus
Th	T-helper
TNF	Tumor necrosis factor
TTFL	Transcription-translation feedback loop

## References

[1] Doyle S., Menaker M. (2007). Circadian photoreception in vertebrates. Cold Spring Harb. Symp. Quant. Biol..

[2] Jones C.R., Huang A.L., Ptáček L.J., Fu Y.H. (2013). Genetic basis of human circadian rhythm disorders. Exp. Neurol..

[3] Sherratt M.J., Hopkinson L., Naven M., Hibbert S.A., Ozols M., Eckersley A., Newton V.L., Bell M., Meng Q.J. (2019). Circadian rhythms in skin and other elastic tissues. Matrix Biol..

[4] Hastings M.H., Reddy A.B., Maywood E.S. (2003). A clockwork web: Circadian timing in brain and periphery, in health and disease. Nat. Rev. Neurosci..

[5] Dyar K.A., Lutter D., Artati A., Ceglia N.J., Liu Y., Armenta D., Jastroch M., Schneider S., De Mateo S., Cervantes M. (2018). Atlas of circadian metabolism reveals system-wide coordination and communication between clocks. Cell.

[6] Partch C.L., Green C.B., Takahashi J.S. (2014). Molecular architecture of the mammalian circadian clock. Trends Cell Biol..

[7] Pett J.P., Kondoff M., Bordyugov G., Kramer A., Herzel H. (2018). Co-existing feedback loops generate tissue-specific circadian rhythms. Life Sci. Alliance.

[8] Huang N., Chelliah Y., Shan Y., Taylor C.A., Yoo S.H., Partch C., Green C.B., Zhang H., Takahashi J.S. (2012). Crystal structure of the heterodimeric CLOCK: BMAL1 transcriptional activator complex. Science.

[9] Takahashi J.S. (2017). Transcriptional architecture of the mammalian circadian clock. Nat. Rev. Genet..

[10] Lipton J.O., Yuan E.D., Boyle L.M., Ebrahimi-Fakhari D., Kwiatkowski E., Nathan A., Güttler T., Davis F., Asara J.M., Sahin M. (2015). The circadian protein BMAL1 regulates translation in response to S6K1-mediated phosphorylation. Cell.

[11] Michael A.K., Asimgil H., Partch C.L. (2015). Cytosolic BMAL1 moonlights as a translation factor. Trends Biochem. Sci..

[12] Asher G., Gatfield D., Stratmann M., Reinke H., Dibner C., Kreppel F., Mostoslavsky R., Alt F.W., Schibler U. (2008). SIRT1 regulates circadian clock gene expression through PER2 deacetylation. Cell.

[13] Eide E.J., Woolf M.F., Kang H., Woolf P., Hurst W., Camacho F., Vielhaber E.L., Giovanni A., Virshup D.M. (2005). Control of mammalian circadian rhythm by CKIepsilon-regulated proteasome-mediated PER2 degradation. Mol. Cell Biol..

[14] Doi M., Hirayama J., Sassone-Corsi P. (2006). Circadian regulator CLOCK is a histone acetyltransferase. Cell.

[15] Ukai-Tadenuma M., Yamada R.G., Xu H., Ripperger J.A., Liu A.C., Ueda H.R. (2011). Delay in feedback repression by cryptochrome 1 is required for circadian clock function. Cell.

[16] Lee C., Etchegaray J.P., Cagampang F.R., Loudon A.S., Reppert S.M. (2001). Posttranslational mechanisms regulate the mammalian circadian clock. Cell.

[17] Takahashi J.S., Hong H.K., Ko C.H., McDearmon E.L. (2008). The genetics of mammalian circadian order and disorder: Implications for physiology and disease. Nat. Rev. Genet..

[18] Cao R., Gkogkas C.G., de Zavalia N., Blum I.D., Yanagiya A., Tsukumo Y., Xu H., Lee C., Storch K.F., Liu A.C. (2015). Light-regulated translational control of circadian behavior by eIF4E phosphorylation. Nat. Neurosci..

[19] Lee H.M., Chen R., Kim H., Etchegaray J.P., Weaver D.R., Lee C. (2011). The period of the circadian oscillator is primarily determined by the balance between casein kinase 1 and protein phosphatase 1. Proc. Natl. Acad. Sci. USA.

[20] Bertolotti A. (2018). The split protein phosphatase system. Biochem. J..

[21] Partch C.L., Shields K.F., Thompson C.L., Selby C.P., Sancar A. (2006). Posttranslational regulation of the mammalian circadian clock by cryptochrome and protein phosphatase 5. Proc. Natl. Acad. Sci. USA.

[22] Guo G., Wang K., Hu S.S., Tian T., Liu P., Mori T., Chen P., Johnson C.H., Qin X. (2019). Autokinase activity of casein kinase 1 δ/ε governs the period of mammalian circadian rhythms. J. Biol. Rhythm..

[23] Skinner J., Sinclair C., Romeo C., Armstrong D., Charbonneau H., Rossie S. (1997). Purification of a fatty acid-stimulated protein-serine/threonine phosphatase from bovine brain and its identification as a homolog of protein phosphatase 5. J. Biol. Chem..

[24] Dobson S., Kar B., Kumar R., Adams B., Barik S. (2001). A novel tetratricopeptide repeat (TPR) containing PP5 serine/threonine protein phosphatase in the malaria parasite, *Plasmodium falciparum*. BMC Microbiol..

[25] Egli M., Johnson C.H. (2013). A circadian clock nanomachine that runs without transcription or translation. Curr. Opin. Neurobiol..

[26] Heath-Heckman E.A.C. (2016). The metronome of symbiosis: Interactions between microbes and the host circadian clock. Integr. Comp. Biol..

[27] Labrecque N., Cermakian N. (2015). Circadian clocks in the immune system. J. Biol. Rhythm..

[28] Nakao A. (2014). Temporal regulation of cytokines by the circadian clock. J. Immunol. Res..

[29] Tsoumtsa L.L., Torre C., Ghigo E. (2016). Circadian control of antibacterial immunity: Findings from animal models. Front. Cell. Infect. Microbiol..

[30] Tognini P., Thaiss C.A., Elinav E., Sassone-Corsi P. (2017). Circadian coordination of antimicrobial responses. Cell Host Microbe.

[31] Westwood M.L., O’Donnell A.J., de Bekker C., Lively C.M., Zuk M., Reece S.E. (2019). The evolutionary ecology of circadian rhythms in infection. Nat. Ecol. Evol..

[32] Panda S., Antoch M.P., Miller B.H., Su A.I., Schook A.B., Straume M., Schultz P.G., Kay S.A., Takahashi J.S., Hogenesch J.B. (2002). Coordinated transcription of key pathways in the mouse by the circadian clock. Cell.

[33] Storch K.F., Lipan O., Leykin I., Viswanathan N., Davis F.C., Wong W.H., Weitz C.J. (2002). Extensive and divergent circadian gene expression in liver and heart. Nature.

[34] Miller B.H., McDearmon E.L., Panda S., Hayes K.R., Zhang J., Andrews J.L., Antoch M.P., Walker J.R., Esser K.A., Hogenesch J.B. (2007). Circadian and CLOCK-controlled regulation of the mouse transcriptome and cell proliferation. Proc. Natl. Acad. Sci. USA.

[35] McCarthy J.J., Andrews J.L., McDearmon E.L., Campbell K.S., Barber B.K., Miller B.H., Walker J.R., Hogenesch J.B., Takahashi J.S., Esser K.A. (2007). Identification of the circadian transcriptome in adult mouse skeletal muscle. Physiol. Genom..

[36] Hatanaka F., Matsubara C., Myung J., Yoritaka T., Kamimura N., Tsutsumi S., Kanai A., Suzuki Y., Sassone-Corsi P., Aburatani H. (2010). Genome-wide profiling of the core clock protein BMAL1 targets reveals a strict relationship with metabolism. Mol. Cell. Biol..

[37] Korenčič A., Košir R., Bordyugov G., Lehmann R., Rozman D., Herzel H. (2014). Timing of circadian genes in mammalian tissues. Sci. Rep..

[38] Atger F., Gobet C., Marquis J., Martin E., Wang J., Weger B., Lefebvre G., Descombes P., Naef F., Gachon F. (2015). Circadian and feeding rhythms differentially affect rhythmic mRNA transcription and translation in mouse liver. Proc. Natl. Acad. Sci. USA.

[39] Sato S., Solanas G., Peixoto F.O., Bee L., Symeonidi A., Schmidt M.S., Brenner C., Masri S., Benitah S.A., Sassone-Corsi P. (2017). Circadian reprogramming in the liver identifies metabolic pathways of aging. Cell.

[40] Zhang Z., Hunter L., Wu G., Maidstone R., Mizoro Y., Vonslow R., Fife M., Hopwood T., Begley N., Saer B. (2019). Genome-wide effect of pulmonary airway epithelial cell-specific Bmal1 deletion. FASEB J..

[41] Bunger M.K., Wilsbacher L.D., Moran S.M., Clendenin C., Radcliffe L.A., Hogenesch J.B., Simon M.C., Takahashi J.S., Bradfield C.A. (2000). Mop3 is an essential component of the master circadian pacemaker in mammals. Cell.

[42] Edgar R.S., Stangherlin A., Nagy A.D., Nicoll M.P., Efstathiou S., O’Neill J.S., Reddy A.B. (2016). Cell autonomous regulation of herpes and influenza virus infection by the circadian clock. Proc. Natl. Acad. Sci. USA.

[43] Majumdar T., Dhar J., Patel S., Kondratov R., Barik S. (2017). Circadian transcription factor BMAL1 regulates innate immunity against select RNA viruses. Innate Immun..

[44] Zhuang X., Rambhatla S.B., Lai A.G., McKeating J.A. (2017). Interplay between circadian clock and viral infection. J. Mol. Med..

[45] Sherling D.H., Perumareddi P., Hennekens C.H. (2017). Metabolic Syndrome. J. Cardiovasc. Pharmacol. Ther..

[46] Ko C.H., Takahashi J.S. (2006). Molecular components of the mammalian circadian clock. Hum. Mol. Genet..

[47] Engin A. (2017). Circadian rhythms in diet-induced obesity. Adv. Exp. Med. Biol..

[48] Sartor F., Eelderink-Chen Z., Aronson B., Bosman J., Hibbert L.E., Dodd A.N., Kovács Á.T., Merrow M. (2019). Are there circadian clocks in non-photosynthetic bacteria?. Biology.

[49] Loza-Correa M., Gomez-Valero L., Buchrieser C. (2010). Circadian clock proteins in prokaryotes: Hidden rhythms?. Front. Microbiol..

[50] Barko P.C., McMichael M.A., Swanson K.S., Williams D.A. (2018). The gastrointestinal microbiome: A Review. J. Vet. Intern. Med..

[51] Kaper J.B., Nataro J.P., Mobley H.L. (2004). Pathogenic *Escherichia coli*. Nat. Rev. Microbiol..

[52] Leimbach A., Hacker J., Dobrindt U. (2013). E. *coli* as an all-rounder: The thin line between commensalism and pathogenicity. Curr. Top. Microbiol. Immunol..

[53] Bengoechea J.A., Sa Pessoa J. (2019). *Klebsiella pneumoniae* infection biology: Living to counteract host defences. FEMS Microbiol. Rev..

[54] Xia J., Gao J., Tang W. (2016). Nosocomial infection and its molecular mechanisms of antibiotic resistance. Biosci. Trends..

[55] Simon A.K., Hollander G.A., McMichael A. (2015). Evolution of the immune system in humans from infancy to old age. Proc. R. Soc. B Biol. Sci..

[56] Kumar R., Ison M.G. (2019). Opportunistic infections in transplant patients. Infect. Dis. Clin..

[57] Van Gastel A. (2018). Drug-induced insomnia and excessive sleepiness. Sleep Med. Clin..

[58] Novak M., Shapiro C.M. (1997). Drug-induced sleep disturbances. Focus on nonpsychotropic medications. Drug Saf..

[59] Thaiss C.A., Zmora N., Levy M., Elinav E. (2016). The microbiome and innate immunity. Nature.

[60] Fabbrizzi A., Amedei A., Lavorini F., Renda T., Fontana G. (2019). The lung microbiome: Clinical and therapeutic implications. Intern. Emerg. Med..

[61] Jandhyala S.M., Talukdar R., Subramanyam C., Vuyyuru H., Sasikala M., Reddy D.N. (2015). Role of the normal gut microbiota. World J. Gastroenterol..

[62] Nagpal R., Wang S., Ahmadi S., Hayes J., Gagliano J., Subashchandrabose S., Kitzman D.W., Becton T., Read R., Yadav H. (2018). Human-origin probiotic cocktail increases short-chain fatty acid production via modulation of mice and human gut microbiome. Sci. Rep..

[63] Das P., Babaei P., Nielsen J. (2019). Metagenomic analysis of microbe-mediated vitamin metabolism in the human gut microbiome. BMC Genom..

[64] Lewis J.D., Chen E.Z., Baldassano R.N., Otley A.R., Griffiths A.M., Lee D., Bittinger K., Bailey A., Friedman E.S., Hoffmann C. (2015). Inflammation, antibiotics, and diet as environmental stressors of the gut microbiome in pediatric Crohn’s disease. Cell Host Microbe.

[65] Voigt R.M., Forsyth C.B., Green S.J., Mutlu E., Engen P., Vitaterna M.H., Turek F.W., Keshavarzian A. (2014). Circadian disorganization alters intestinal microbiota. PLoS ONE.

[66] Buxton O.M., Cain S.W., O’Connor S.P., Porter J.H., Duffy J.F., Wang W., Czeisler C.A., Shea S.A. (2012). Adverse metabolic consequences in humans of prolonged sleep restriction combined with circadian disruption. Sci. Transl. Med..

[67] Fonken L.K., Workman J.L., Walton J.C., Weil Z.M., Morris J.S., Haim A., Nelson R.J. (2010). Light at night increases body mass by shifting the time of food intake. Proc. Natl. Acad. Sci. USA.

[68] Scheer F.A., Hilton M.F., Mantzoros C.S., Shea S.A. (2009). Adverse metabolic and cardiovascular consequences of circadian misalignment. Proc. Natl. Acad. Sci. USA.

[69] Paulose J.K., Wright J.M., Patel A.G., Cassone V.M. (2016). Human gut bacteria are sensitive to melatonin and express endogenous circadian rhythmicity. PLoS ONE.

[70] Roach G.D., Sargent C. (2019). Interventions to minimize jet lag after westward and eastward flight. Front. Physiol..

[71] Süel G.M., Garcia-Ojalvo J., Liberman L.M., Elowitz M.B. (2006). An excitable gene regulatory circuit induces transient cellular differentiation. Nature.

[72] Stroppa M.M., Gimenez I., García B.A. (2018). Clock gene Period in the Chagas disease vector *Triatoma infestans* (Hemiptera: *Reduviidae*). Am. J. Trop. Med. Hyg..

[73] Kratz J.M. (2019). Drug discovery for chagas disease: A viewpoint. Acta Trop..

[74] Rijo-Ferreira F., Pinto-Neves D., Barbosa-Morais N.L., Takahashi J.S., Figueiredo L.M. (2017). *Trypanosoma brucei* metabolism is under circadian control. Nat. Microbiol..

[75] Narula A.K., Azad C.S., Nainwal L.M. (2019). New dimensions in the field of antimalarial research against malaria resurgence. Eur. J. Med. Chem..

[76] Marques M.D. (2013). Biological rhythms and vector insects. Mem. Inst. Oswaldo Cruz.

[77] O’Donnell A.J., Schneider P., McWatters H.G., Reece S.E. (2011). Fitness costs of disrupting circadian rhythms in malaria parasites. Proc. R. Soc. B Biol. Sci..

[78] Njamnshi A.K., Gettinby G., Kennedy P.G.E. (2017). The challenging problem of disease staging in human African trypanosomiasis (sleeping sickness): A new approach to a circular question. Trans. R. Soc. Trop. Med. Hyg..

[79] Tesoriero C., Xu Y.Z., Mumba Ngoyi D., Bentivoglio M. (2018). Neural damage in experimental *Trypanosoma brucei gambiense* infection: The suprachiasmatic nucleus. Front. Neuroanat..

[80] Lyczak J.B., Cannon C.L., Pier G.B. (2002). Lung infections associated with cystic fibrosis. Clin. Microbiol. Rev..

[81] Zaoutis T.E., Argon J., Chu J., Berlin J.A., Walsh T.J., Feudtner C. (2005). The epidemiology and attributable outcomes of candidemia in adults and children hospitalized in the United States: A propensity analysis. Clin. Infect. Dis..

[82] Hevia M.A., Canessa P., Larrondo L.F. (2016). Circadian clocks and the regulation of virulence in fungi: Getting up to speed. Semin. Cell Dev. Biol..

[83] Brody S. (2019). Circadian rhythms in fungi: Structure/function/evolution of some clock components. J. Biol. Rhythm..

[84] Salichos L., Rokas A. (2010). The diversity and evolution of circadian clock proteins in fungi. Mycologia.

[85] Spearman C.W., Dusheiko G.M., Hellard M., Sonderup M. (2019). Hepatitis C. Lancet.

[86] Vinciguerra M., Mazzoccoli G., Piccoli C., Tataranni T., Andriulli A., Pazienza V. (2013). Exploitation of host clock gene machinery by hepatitis viruses B and C. World J. Gastroenterol..

[87] Jopling C.L., Schutz S., Sarnow P. (2008). Position-dependent function for a tandem microRNA miR-122-binding site located in the hepatitis C virus RNA genome. Cell Host Microbe.

[88] Gatfield D., Le Martelot G., Vejnar C.E., Gerlach D., Schaad O., Fleury-Olela F., Ruskeepaa A.L., Oresic M., Esau C.C., Zdobnov E.M. (2009). Integration of microRNA miR-122 in hepatic circadian gene expression. Genes Dev..

[89] Zhuang X., Magri A., Hill M., Lai A.G., Kumar A., Rambhatla S.B., Donald C.L., Lopez-Clavijo A.F., Rudge S., Pinnick K. (2019). The circadian clock components BMAL1 and REV-ERBα regulate flavivirus replication. Nat. Commun..

[90] Lyu J., Imachi H., Fukunaga K., Yoshimoto T., Zhang H., Murao K. (2015). Roles of lipoprotein receptors in the entry of hepatitis C virus. World J. Hepatol..

[91] Martín-Acebes M.A., Jiménez de Oya N., Saiz J.C. (2019). Lipid metabolism as a source of druggable targets for antiviral discovery against Zika and other flaviviruses. Pharmaceuticals.

[92] Ehlers A., Xie W., Agapov E., Brown S., Steinberg D., Tidwell R., Sajol G., Schutz R., Weaver R., Yu H. (2017). BMAL1 links the circadian clock to viral airway pathology and asthma phenotypes. Mucosal Immunol..

[93] Kalamvoki M., Roizman B. (2011). The histone acetyltransferase CLOCK is an essential component of the herpes simplex virus 1 transcriptome that includes TFIID, ICP4, ICP27, and ICP22. J. Virol..

[94] Jenkins P.J., Binné U.K., Farrell P.J. (2000). Histone acetylation and reactivation of Epstein-Barr virus from latency. J. Virol..

[95] Murata T. (2014). Regulation of Epstein-Barr virus reactivation from latency. Microbiol. Immunol..

[96] Lumbreras C., Colina F., Loinaz C., Domingo M.J., Fuertes A., Dominguez P., Gómez R., Aguado J.M., Lizasoain M., González-Pinto I. (1998). Clinical, virological, and histologic evolution of hepatitis C virus infection in liver transplant recipients. Clin. Infect. Dis..

[97] Garcia-Retortillo M., Forns X., Feliu A., Moitinho E., Costa J., Navasa M., Rimola A., Rodes J. (2002). Hepatitis C virus kinetics during and immediately after liver transplantation. Hepatology.

[98] Benegiamo G., Mazzoccoli G., Cappello F., Rappa F., Scibetta N., Oben J., Greco A., Williams R., Andriulli A., Vinciguerra M. (2013). Mutual antagonism between circadian protein period 2 and hepatitis C virus replication in hepatocytes. PLoS ONE.

[99] Sundar I.K., Ahmad T., Yao H., Hwang J.W., Gerloff J., Lawrence B.P., Sellix M.T., Rahman I. (2015). Influenza A virus-dependent remodeling of pulmonary clock function in a mouse model of COPD. Sci. Rep..

[100] Gibbs J.E., Blaikley J., Beesley S., Matthews L., Simpson K.D., Boyce S.H., Farrow S.N., Else K.J., Singh D., Ray D.W. (2012). The nuclear receptor REV-ERBα mediates circadian regulation of innate immunity through selective regulation of inflammatory cytokines. Proc. Natl. Acad. Sci. USA.

[101] Gibbs J., Ince L., Matthews L., Mei J., Bell T., Yang N., Saer B., Begley N., Poolman T., Pariollaud M. (2014). An epithelial circadian clock controls pulmonary inflammation and glucocorticoid action. Nat. Med..

[102] Keller M., Mazuch J., Abraham U., Eom G.D., Herzog E.D., Volk H.D., Kramer A., Maier B. (2009). A circadian clock in macrophages controls inflammatory immune responses. Proc. Natl. Acad. Sci. USA.

[103] Bellet M.M., Deriu E., Liu J.Z., Grimaldi B., Blaschitz C., Zeller M., Edwards R.A., Sahar S., Dandekar S., Baldi P. (2013). Circadian clock regulates the host response to *Salmonella*. Proc. Natl. Acad. Sci. USA.

[104] Richard J.L., Sotto A., Lavigne J.P. (2011). New insights in diabetic foot infection. World J. Diabetes.

[105] Kiessling S., Dubeau-Laramée G., Ohm H., Labrecque N., Olivier M., Cermakian N. (2017). The circadian clock in immune cells controls the magnitude of *Leishmania* parasite infection. Sci. Rep..

[106] Hopwood T.W., Hall S., Begley N., Forman R., Brown S., Vonslow R., Saer B., Little M.C., Murphy E.A., Hurst R.J. (2018). The circadian regulator BMAL1 programmes responses to parasitic worm infection via a dendritic cell clock. Sci. Rep..

[107] Yu X., Rollins D., Ruhn K.A., Stubblefield J.J., Green C.B., Kashiwada M., Rothman P.B., Takahashi J.S., Hooper L.V. (2013). TH17 cell differentiation is regulated by the circadian clock. Science.

[108] Phillips A.C., Gallagher S., Carroll D., Drayson M. (2008). Preliminary evidence that morning vaccination is associated with an enhanced antibody response in men. Psychophysiology.

[109] Kirby T. (2016). Influenza vaccination in the morning improves response. Lancet Respir. Med..

[110] Long J.E., Drayson M.T., Taylor A.E., Toellner K.M., Lord J.M., Phillips A.C. (2016). Morning vaccination enhances antibody response over afternoon vaccination: A cluster-randomised trial. Vaccine.

[111] Nakahata Y., Yoshida M., Takano A., Soma H., Yamamoto T., Yasuda A., Nakatsu T., Takumi T. (2008). A direct repeat of E-box-like elements is required for cell-autonomous circadian rhythm of clock genes. BMC Mol. Biol..

[112] Lieu P.T., Wagner E.K. (2000). Two leaky-late HSV-1 promoters differ significantly in structural architecture. Virology.

[113] Romani B., Allahbakhshi E. (2017). Underlying mechanisms of HIV-1 latency. Virus Genes.

[114] Shirakawa K., Chavez L., Hakre S., Calvanese V., Verdin E. (2013). Reactivation of latent HIV by histone deacetylase inhibitors. Trends Microbiol..

[115] Sigworth L.A., Liao L., Chandler T.R., Geusz M.E. (2003). Luciferase expression controlled by a viral gene promoter in a mammalian circadian pacemaker. Neuroreport.

[116] Sothern R.B., Cornélissen G., Yamamoto T., Takumi T., Halberg F. (2009). Time microscopy of circadian expression of cardiac clock gene mRNA transcription: Chronodiagnostic and chrono-therapeutic implications. Clin. Ter..

[117] Del Olmo M., Kramer A., Herzel H. (2019). A robust model for circadian redox oscillations. Int. J. Mol. Sci..

[118] Hughes M.E., Abruzzi K.C., Allada R., Anafi R., Arpat A.B., Asher G., Baldi P., de Bekker C., Bell-Pedersen D., Blau J. (2017). Guidelines for genome-scale analysis of biological rhythms. J. Biol. Rhythm..

[119] Taylor G. (2017). Animal models of respiratory syncytial virus infection. Vaccine.

[120] Daugherty M.D., Schaller A.M., Geballe A.P., Malik H.S. (2016). Evolution-guided functional analyses reveal diverse antiviral specificities encoded by *IFIT1* genes in mammals. Elife.

[121] Lavappa K.S. (1978). Survey of ATCC stocks of human cell lines for HeLa contamination. In Vitro.

[122] Nikhil K.L., Korge S., Achim K. (2019). Circadian period-heterogeneity is governed by clonal inheritance of variable gene expression. BioRixv.

[123] Bosman J., Eelderink-Chen Z., Laing E., Merrow M. (2018). PREMONition: An algorithm for predicting the circadian clock-regulated molecular network. BioRixv.

